# Therapeutic efficacy of high-dose chemotherapy with autologous stem-cell transplantation in 44 relapsed or refractory germ-cell tumor patients: A retrospective cohort study

**DOI:** 10.1097/MD.0000000000037213

**Published:** 2024-02-23

**Authors:** Ferhat Ferhatoglu, Nail Paksoy, Nijat Khanmammadov, Anil Yildiz, Melin Aydan Ahmed, Zafer Gülbas, Mert Basaran

**Affiliations:** a Department of Medical Oncology, Basaksehir Cam and Sakura City Hospital, Istanbul, Turkey; b Department of Medical Oncology, Istanbul University Institute of Oncology, Istanbul, Turkey; c Bone Marrow Transplantation Center, Anadolu Medical Center, Kocaeli, Turkey

**Keywords:** germ-cell tumor, high-dose chemotherapy, prognosis, survival

## Abstract

Despite having a higher mortality risk than conventional chemotherapeutics, high-dose chemotherapy (HDCT) has the potential to be curative in relapsed/refractory germ-cell tumors. Therefore, selecting the best patient group for this treatment is critical. This study aimed to determine the factors that affect survival in our relapsed/refractory GCT cohort who received HDCT and autologous stem-cell transplantation. Between September 2010 and 2020, we included in the study 44 relapsed/refractory male patients with GCT treated with HDCT plus autologous stem-cell transplantation. The patients’ demographic features, clinical characteristics, and treatment outcomes were evaluated. Statistical analyses were performed to identify risk factors associated with survival. The median age of all cohorts was 28 years. Thirty-six patients had nonseminomatous tumors, and 8 patients had seminomatous tumors. The most common primary tumor sites were the gonads (75%), followed by the mediastinum (15.9%) and the retroperitoneum (9.1%). After HDCT, 11 patients had a complete response, 12 patients had a partial response, and 17 patients had a progressive disease, respectively. About 23 patients (52.3%) experienced at least 1 treatment-related grade 3 to 4 nonhematological toxicity. About 4 patients (10%) died due to HDCT-related toxicity. The total group’s median progression-free survival (PFS) was 7 months, and the median overall survival (OS) was 14.9 months. Primary tumor site (hazard ratio [HR]: 1.84; *P* = .028), type of HDCT regimen (HR: 0.35; *P* = .010), and best response to HDCT (HR: 11.0; *P* < .0001) were independent prognostic risk factors for PFS. The only independent prognostic risk factor associated with OS was the best response to HDCT (HR: 6.62; *P* = .001). The results of the study promise the best response to HDCT as a primary measure for predicting survival in relapsed/refractory GCT. In contrast, primary mediastinal GCT is not a good candidate for HDCT. Furthermore, a carboplatin–etoposide regimen in combination with cyclophosphamide and paclitaxel may improve PFS.

## 1. Introduction

Germ-cell tumors, which have a cure potential even in distant metastases, are often diagnosed in young and young–middle-aged patients.^[1]^ With cisplatin and etoposide (EP) or adding bleomycin to this combination (BEP), up to 80% of cure can be achieved in metastatic disease.^[2]^ Nevertheless, the International Germ-Cell Cancer Collaborative Group (IGCCCG) stratified metastatic disease into 3 risk groups in a large retrospective study to optimize initial therapy based on a more accurate prognosis prediction.^[3]^ The 5-years overall survival (OS) rates in this study were 91% (good prognosis), 79% (intermediate prognosis), and 48% (poor prognosis).

However, no consensus has been reported on the best salvage therapy in relapsed/refractory disease, which accounts for 30% of patients with GCT.^[4]^ Historically, 2 main salvage treatment approaches have emerged. The first is conventional-dose chemotherapy treatments, such as paclitaxel–ifosfamide–cisplatin (TIP) and vinblastine–ifosfamide–cisplatin (VeIP), which contain cisplatin similar to the initial treatment. Despite being distinct chemotherapeutics, paclitaxel and vinorelbine can act on cellular microtubules. In prospective studies, TIP and VeIP had complete response (CR) rates of 70% and 49.9%, respectively.^[5,6]^ However, there is no direct comparison of the 2 regimens.

High-dose chemotherapy treatment (HDCT) with autologous stem-cell transplantation (ASCT) is the last option for relapsed/refractory GCT. The rationale of the context has gained prominence after experimental studies that found a positive correlation between chemotherapy doses and tumor responses. In the 1970s, HDCT was shown to be an effective treatment for relapsed hematological malignancies.^[7,8]^ After promising pioneering studies, HDCT became a salvage treatment option in the following years.^[9,10]^ Nichols et al conducted a phase 1 to 2 study in 1989 in which 33 patients with extensively pretreated or cisplatin refractory GCT were given a high-dose carboplatin–etoposide (CE) combination. They found CR in 7 (25%) patients, with 3 CRs lasting more than 1 year. In this study, treatment-related mortality was 25%.^[11]^ Other retrospective studies found that high-dose CE combination resulted in 11 CR rates ranging from 30% to 63%.^[12,13]^ Lorch et al^[14]^ evaluated the efficiency of high-dose CE combination vis-à-vis CDCT in patients with relapsed/refractory GCT; both progression-free survival (PFS) (hazard ratio [HR]: 0.44) and OS (HR: 0.65) were better in the HDCT arm than the CDCT. However, no phase 3 randomized study has shown HDCT to be better than CDCT. Because the fundamentals of HDCT are platinum-based compared with CDCT, the frequency and higher grades of toxicities are significantly higher than in CDCT. Despite its complex applications and toxicity profile, HDCT has become a salvage treatment option for patients with relapsed/refractory GCT due to its ability to provide a sustained response and a cure probability.

Specific prognostic models were also developed to predict salvage treatment outcomes in patients with relapsed/refractory GCT. Primary tumor site, response to previous platinum-based therapy, IGCCCG risk score, 2 or more chemotherapy lines before HDCT, and serum tumor marker levels (B-HCG and AFP) are major prognostic factors in these models.^[13,15–17]^ However, it should be noted that the patient groups represented in these models showed heterogeneity among studies.

We needed to examine the prognostic factors of our patients with relapsed/refractory GCT who received HDCT and compare the efficacy of different HDCT regimens in this context to contribute to the relevant problem.

## 2. Material and methods

### 2.1. Study protocol and patient selection criteria

The design of the study was based on a retrospective cohort study. First, between September 2010 and 2020, the clinical data of 1254 patients with a pathologically confirmed diagnosis of GCT based on previous biopsies or surgical materials were retrospectively scanned at the medical oncology departments at Istanbul University Institute of Oncology, a single tertiary referral center. All patients in the study had to meet the following inclusion criteria: male gender, 16 years or older, receiving platinum-based first-line chemotherapy (BEP or EP), refractory to first platinum-based chemotherapy regimen (progression during or within 4 weeks after treatment), relapse 4 weeks after the first platinum-based chemotherapy regimen, receiving second-line platinum-based chemotherapy (the first salvage therapy) either with CDCT or HDCT, receiving at least 1 cycle HDCT with ASCT at second-line or later, and complete demographic, clinic, pathologic, and laboratory data with treatment information, including chemotherapy doses, implication times, and treatment-related toxicities. Patients who did not meet the criteria above were excluded from the study. After selection, there were 44 male patients whose medical records were available for further analysis.

### 2.2. Clinical features, definition of responses, and prognosis evaluation

We classified primary tumor histology as seminoma and nonseminoma. The primary tumor site was evaluated in 3 anatomical regions: gonads, retroperitoneum, and mediastinum. The best response to chemotherapy classified into 3 subgroups (CR, incomplete response [IR], and progressive disease [PD]) was used as a standard response evaluation for first-line chemotherapy, second-line chemotherapy, and HDCT. CR was defined as decreasing B-HCG and AFP levels below the nadir level and the complete disappearance of tumor foci in radiological images. PD was recognized as increased B-HCG and AFP levels immediately after treatment. These 2 responses were classified as IR.

The IGCCCG risk score system was used to evaluate the prognosis based on the disease’s initial diagnosis. Other risk factors included the best response to first-line chemotherapy, a 2-year disease-free interval (from the completion of first-line chemotherapy to the first recurrence), tumor locations at first relapse, the best response to second-line chemotherapy, and the best response to HDCT. Receiving 1 or 2 HDCT courses was considered a risk factor in the prognostic evaluation. Accordingly, the line of HDCT application was also divided into a second line or more or equal to the third line. Moreover, to determine the prognostic importance of a subgroup, the primary refractory patients, defined as PD after first-line chemotherapy, were compared in predictive analysis with the remaining cohort of patients receiving second-line or more or equal to third-line HDCT.

Time to neutrophil count was defined as the number of days between the start of the ASCT application and the absolute neutrophil count reaching 1000/mm^3^. Similarly, time to platelet engraftment was calculated as the number of days between the start of the ASCT application and platelet count reaching 20,000/mm^3^.

### 2.3. Treatment protocols

TIP, iphosphamide–cisplatin–etoposide, vincristine–iphosphamide–cisplatin (VIP), and gemcitabine–oxaliplatin (GEMOX) were used as induction chemotherapy before HDCT. CE, carboplatin–etoposide–cyclophosphamide–paclitaxel (carbo–PEC–taxol), topotecan–thiotepa–carboplatin (TTC), and BCNU-etoposide–cytarabine-melphalan (BEAM) were the HDCT regimens used.

Protocols of HDCT and ASCT are as follows:

- CE: Carboplatin area under the curve (AUC) of 10, etoposide of 600 mg/m^2^, and cyclophosphamide of 50 mg/kg administered on Days 2 to 4. Stem-cell rescue was on Day 0.
- Carbo–PEC–taxol: Paclitaxel of 75 mg/m^2^ on Days 3, 5, and 7; etoposide of 450 mg/m^2^ on Days 3, 5, and 7; carboplatin AUC of 10 on Days 3, 5, and 7; and cyclophosphamide of 60 mg/kg on Days 3 and 5. Stem-cell rescue was on Day 0.
- TTC: Thiotepa of 300 mg/m^2^ on Days 6 to 8; topotecan of 2 mg/m^2^ on Days 4 to 8; and carboplatin AUC of 7 on Days 3 to 5. Stem-cell rescue was on Day 0.
- BEAM: BCNU of 300 mg/m^2^ on Day 7, etoposide of 800 mg/m^2^, cytosine arabinoside of 800 mg/m^2^, and cyclophosphamide of 35 mg/kg on Days 3 to 6. Stem-cell rescue was on Day 0.

### 2.4. Statistical analysis

IBM SPSS Statistics for Windows, Version 25.0 (IBM Corp., Armonk, NY), was used for statistical analysis. Categorical variables in descriptive statistics are expressed as numbers and percentages, whereas numerical variables are expressed as median, standard deviation, and minimum and maximum values. Proportions in independent groups were analyzed using the Chi-square test. Numerical variables in 2 independent groups were compared using the Mann–Whitney *U* test or independent *t* test.

PFS and OS were the primary targets of the survival analysis. The completion date of HDCT with ASCT protocol was used as the starting point for PFS and OS. The biochemical or radiological progression of the disease was used as the endpoint for PFS. In terms of OS, the endpoint was the patient’s disease-related death or the date of the last visit. Survival rates were calculated using the Kaplan–Meier analysis. The log-rank test was used to determine statistical differences between subgroups. We used a univariate Cox regression model to estimate hazard ratios (HRs) among subgroups in addition to the Kaplan–Meier analysis. We expanded the Cox regression to the multivariate model in determining independent prognostic factors by integrating outcome-associated covariates after calculating univariate HRs. The statistical significance level of *α* was set at *P* < .05.

## 3. Results

### 3.1. Demographic and clinical features

The study included 44 male patients. The median age at diagnosis was 28 ± 9 years (range: 16–51). About 36 patients (81.8%) had nonseminomatous disease, whereas 8 (18.2%) patients had seminomatous disease. The gonads (75%) were the most prevalent primary tumor site, followed by the mediastinum (15.9%) and the retroperitoneum (9.1%). According to the IGCCCG, the risk scores were approximately evenly distributed; the percentages of good, intermediate, and poor risk groups were 29.5%, 36.4%, and 34.1%, respectively. In terms of first-line chemotherapy response rates, the IR, PD, and CR were 50%, 27.3%, and 22.7%, respectively. The disease-free interval was 13.2 ± 59.8 months (3.61–251.9 months). Furthermore, it lasted more than 2 years for 17 (38.6%) patients. The 3 most prevalent relapse sites at initial recurrence were retroperitoneum (61.4%), lung (34.1%), and mediastinum (15.9%). After the first salvage therapy, 38.6% of patients had PD, 34.1% had IR, and 27.3% had CR (Table 1).

**Table 1:** Characteristics of patients at initial presentation and first relapse features.

Characteristics	Number of patients (*N* = 44)	Percentage (%)
Gender		
Male	44	100
Histology		
Nonseminoma	36	81.8
Seminoma	8	18.2
Age at diagnosis (median age** **± SD; min-max)	28 ± 9	16–51
Primary tumor site		
Gonadal	33	75.0
Retroperitoneum	4	9.10
Mediastinum or other sites	7	15.9
IGCCCG risk score		
Good	13	29.5
Intermediate	16	36.4
Poor	15	34.1
Best response to first-line treatment		
Complete response	10	22.7
Incomplete response	22	50.0
Progressive disease	12	27.3
Disease-free interval		
≤2 years	27	61.4
>2 years	17	38.6
Tumor locations at first relapse		
Retroperitoneum	27	61.4
Lung	15	34.1
Mediastinum	7	15.9
Liver	2	4.5
Brain	1	2.3
Bone	2	4.5
Kidney	3	6.8
Other sites	2	4.5
Best response to second-line treatment		
Complete response	12	27.3
Incomplete response	15	34.1
Progressive disease	17	38.6

Of all patients, 17 (38.6%) received second-line HDCT, and 27 (61.4%) had more or equal to third-line HDCT. The patients with primary refractory PD after first-line chemotherapy were compared with the other patients with HDCT. In this case, the number of patients with primary refractory undergoing second-line HDCT and more or equal to third-line HDCT was 12, 14, and 14, respectively. Nineteen (43.2%) 1 course and 25 (56.8%) 2 courses of HDCT were administered to the patients.

The TIP regimen was the most common type of induction chemotherapy (61.4%). The most common HDCT regimen was CE (68.2%), followed by carbo–PEC–taxol (20.5%), TTC (9.1%), and BEAM (2.3%). The number of CR, IR, and PD after HDCT application was 11 (27.5%), 12 (30.0%), and 17 (42.5%), respectively. Because 4 patients died due to HDCT-related toxicity, a response assessment could not be performed on these patients. The time to neutrophil and platelet engraftment was 9.8 ± 1.8 days (8–19 days) and 12.1 ± 3.8 days (7–23 days), respectively (Table 2).

**Table 2:** High-dose chemotherapy features of the patients.

HDCT features	Number of patients	Percentage (%)
HDCT line		
2nd-line	17	38.6
≥3rd-line	27	61.4
HDCT course		
1	19	43.2
2	25	56.8
Induction chemotherapy regiments		
TIP	27	61.4
ICE	9	20.5
VIP	5	11.4
GEMOX	3	6.8
HDCT regiments		
CE	30	68.2
Carbo–PEC–taxol	9	20.5
TTC	4	9.1
BEAM	1	2.3
Time to neutrophil engraftment (median age** **± SD; min-max)	9.8 ± 1.8	8–19
Time to platelet engraftment (median age** **± SD; min-max)	12.1 ± 3.8	7–23
Best response to HDCT		
Complete response	11	27.5
Incomplete response	12	30.0
Progressive disease	17	42.5

BEAM = BCNU-etoposide-cytarabine-melphalan, Carbo–PEC–taxol = carboplatin–etoposide-cyclophosphamide-paclitaxel, CE = carboplatin–etoposide, HDCT = high-dose chemotherapy, TTC = topotecan–thiotepa-carboplatin.

Twenty-three patients (52.3%) experienced at least 1 treatment-related grade 3 to 4 nonhematological toxicity. In 63 HDCT applications, the most common toxicity was of unknown origin (85.7%). Other common side effects were diarrhea (46.0%), nausea or vomiting (27.0%), and mucositis (22.2%). 4 (10%) patients on Days 7, 11, 16, and 26 died due to HDCT. Engraftment was also not observed in any of these patients. The only cause of HDCT-related mortality was thrombocytopenia, which resulted in intracranial hemorrhage in 2 patients and intratumoral bleeding in 2 patients (Supporting Information Table S1, http://links.lww.com/MD/L730). The frequency of HDCT-related toxicities is shown in Table 3.

**Table 3:** High-dose chemotherapy related toxicities.

Toxicities	Number of events (*N* = 63)	Percentage (%)
Fewer unknown origin	54	85.7
Gram positive sepsis	4	6.3
Gram negative sepsis	3	4.8
Cytomegalovirus sepsis	2	3.2
Candida sepsis	1	1.6
Nausea and vomiting	17	27.0
Diarrhea	29	46.0
Mucositis	14	22.2
Upper gastrointestinal system bleeding	4	6.3
Skin rash	3	4.8
Acute tubular necrosis	2	3.2
Intracranial hemorrhage	1	1.6
Exitus	4	10.0

### 3.2. Survival and prognostic factors

In the entire cohort, the median PFS was 7 months, and the median OS was 14.9 months. Age, tumor histology, IGCCCG score, best response to first-line treatment, disease-free interval, relapse site, and best response to second-line treatment showed no significant effect on PFS and OS in log-rank analysis.

Primary mediastinal GCT showed a worse PFS than retroperitoneal and gonadal GCT (2.2 months vs 5.9 months vs 7.3 months, respectively; *P* = .027). Patients receiving second-line HDCT had considerably longer PFS than those receiving more or equal to third-line HDCT (67.8 months vs 5.9 months; *P* < .0001). Patients on the carbo–PEC–taxol regimen showed a longer PFS than those on CE and TTC (67.8 months vs 7 months vs 2.1 months, respectively; *P* < .0001). After HDCT application, the median PFS for patients with CR was not reached, but it was 9.4 months for IR and 5.4 months for patients with PD (*P* < .0001).

The median OS was 8.7 months in patients with more or equal to third-line HDCT. However, OS did not reach the median time among patients who received second-line HDCT during the study period (*P* < .0001). Patients receiving second-line HDCT and more or equal to third-line HDCT had 2-years survival rates of 79.1% and 19.7%, respectively. When primary refractory patients were compared with the remaining cohort who received second-line and more or equal to third-line HDCT, the 2-years survival rates were 13.8%, 80.0%, and 51.3%, respectively (*P* < .0001).

**Figure 1.**
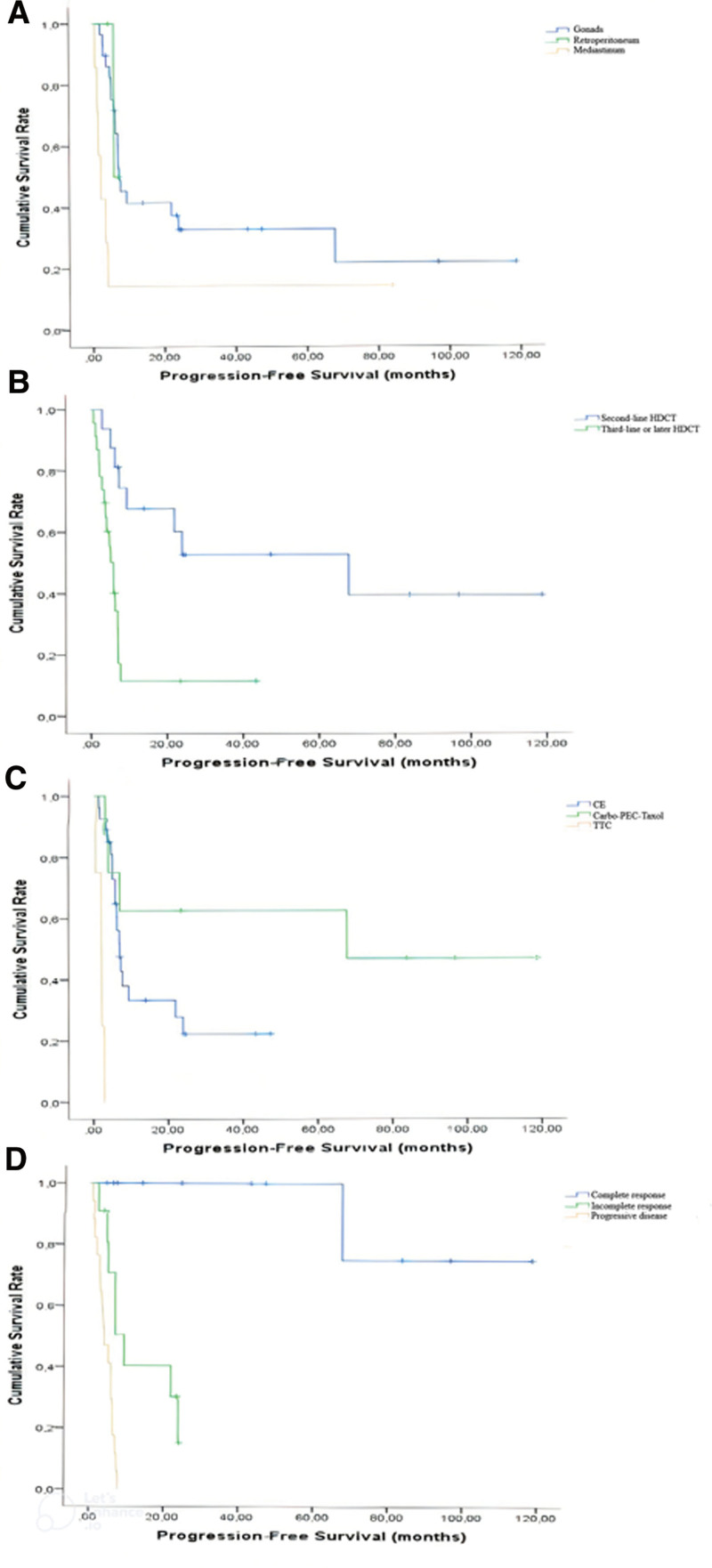
Kaplan–Meier survival curves of prognostic factors related to progression-free survival (PFS); (A) In patients with mediastinal, retroperitoneal, and gonadal germ-cell tumors, PFSs were 2.2 months, 5.9 months, and 7.3 months, respectively (*P* = .027). (B) Patients who received second-line high-dose chemotherapy (HDCT) had significantly longer PFS than those who received third-line or later (67.8 vs 5.9 months; *P* < .0001). (C) Patients receiving Carbo–PEC-Taxol had the longest PFS, followed by CE and TTC (67.8 months vs 7.0 months vs 2.1 months, *P* < .0001). (D) PFS was significantly longer in patients with complete response to HDCT than incomplete response and progressive disease (*P* < .0001).

**Figure 2.**
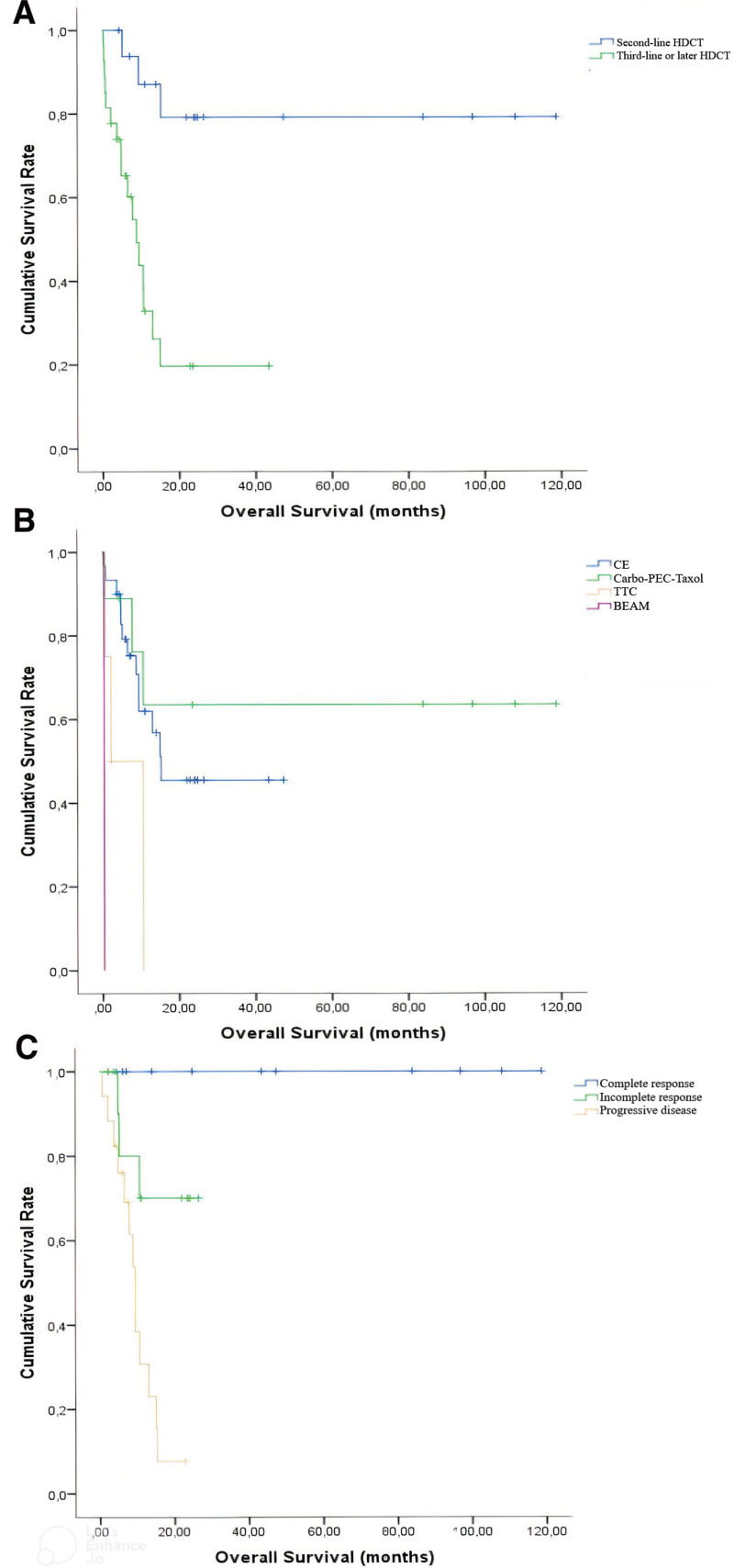
(A) Patients who received second-line high-dose chemotherapy (HDCT) had a more prolonged overall survival (OS) than those who received third-line or later (79.1% vs 19.7%, 2-year survival; *P* < .0001). (B) 2-year survivals were 63.5%, 45.4%, and 0.0% in patients receiving Carbo–PEC-Taxol, CE, and TTC, respectively (*P* = .004). (C) The relationship between response rate and survival was positively correlated (*P* < .0001). 2-year survival was 100% in patients with complete response, 70% in patients with incomplete response, and 7.7% in patients with progressive disease.

The median OS for patients receiving carbo–PEC–taxol was not attained, but it was 34.1 and 9.5 months for CE and TTC, respectively (*P* = .004). Additionally, carbo–PEC–taxol (63.5%) had the highest 2-years OS rate, followed by CE (45.4%) and TTC (0.0%). Based on the best response to HDCT, the 2-years OS rates of CR, IR, and PD were 100%, 70%, and 7.7%, respectively (*P* < .0001). The Kaplan–Meier survival curves for PFS and OS are shown in Figures 1 and 2.

We used the univariate Cox regression model to identify risk factors affected by PFS. Tumor histology (*P* = .244), IGCCC risk score (*P* = .177), best response to first-line treatment (*P* = .095), disease-free interval (*P* = .95), and best response to second-line treatment (*P* = .062) were not identified as significant risk factors in that case. Nonetheless, the primary tumor site (HR: 3.31; 95% confidence interval [95% CI]: 1.28 to 8.53; *P* = .013), HDCT regimen (HR: 0.41; 95% CI, 0.007 to 0.235; *P* < .001), and best response to HDCT (HR: 19.7; 95% CI, 2.43 to 42.2; *P* < .0001) were shown to be significant risk factors for PFS. After that, in multivariate analysis, we found that the primary tumor site (HR: 1.84; 95% CI, 1.06 to 3.19; *P* = .028), HDCT regimen (HR: 0.35; 95% CI, 0.16 to 0.77; *P* = .010), and best response to HDCT (HR: 11.0; 95% CI, 4.19 to 28.9; *P* < .0001) were independent prognostic risk factors (Table 4). In respect of OS, tumor histology (*P* = .95), IGCCC risk score (*P* = .138), best response to first-line treatment (*P *= .077), disease-free interval (*P* = .549), and best response to second-line treatment (*P* = .087) were not identified as univariate risk factors for OS. The primary tumor site (HR: 2.96; 95% CI, 1.03–8.45; *P* = .043), HDCT regimen (HR: 0.195; 95% CI, 0.060–0.637; *P *= .007), and best response to HDCT (HR: 11.2; 95% CI, 3.24–37.8; *P* < .0001) were shown to be significant risk factors for OS. The only independent prognostic risk factor associated with OS was the best response to HDCT (HR: 6.62; 95% CI, 2.18–20.0; *P* = .001).

**Table 4:** Risks factors for progression-free survival, multivariate cox regression analysis.

Multivariate analysis			
Risk factors	P	HR	%95 CI
Primary tumor site	0.028	1.84	1.06–3.19
HDCT regimen	0.010	0.35	0.16–0.77
Best response to HDCT	<0.0001	11.0	4.19–28.9

CI = confidence interval, HDCT = high-dose chemotherapy, HR = hazard ratio.

## 4. Discussion

This study examined the clinical and prognostic factors in patients with relapsed/refractory GCT who had HDCT followed by ACST. Most of our cohort had nonseminomatous histology and were young or young–middle aged. The patients’ IGCCCG risk scores were relatively evenly distributed (Table 1). The median age range and IGCCCG risk group distribution of our patients are comparable with other studies.^[13,14,17,18]^ In this regard, our results are comparable with clinical studies evaluating the efficacy of HDCT. Therefore, we believe the best response to HDCT and the primary tumor site is important when estimating our cohort PFS and OS.

In a study of 40 patients with relapsed/refractory GCT, including patients with extensively pretreated or cisplatin refractory, Broun et al^[12]^ obtained 30% CR with a double-dose CE regimen. Further, 15% had remission for more than 2 years, and the mortality rate was 20% in this study. The CR rate in our study was 30.0%, and the 2- and 5-year PFS rates were 29.8% and 22.3%, respectively. Notably, most patients who obtained a 2-year PSF also had a 5-year PFS. A remission period of more than 2 years may be a criterion for a long-term response. In our study, there was no significant difference in survival between patients who received single or multiple cycles of HDCT. Einhorn et al^[13]^ recently published a retrospective study in which 174 of 184 patients received 2 cycles of a high-dose CE regimen with ASCT support. In 63% of patients, CR lasted 2 years, and 3 (1.6%) died due to treatment-related toxicity.^[13]^ The exclusion of patients with primary mediastinal GCT in this study may have contributed to better response rates than other studies. In the study of Einhorn et al,^[13]^ DFS rates were 69.6% for second-line HDCT and 44.8% for more or equal to third-line HDCT at the end of the 48-month follow-up. According to our results, the 2-year PFS rates in patients who received second-line HDCT and more or equal to third-line HDCT were 52.7% and 11.5%, respectively. In addition, patients who received second-line HDCT had significantly longer PFS and OS (*P* < .0001 for both). HDCT appears to be more promising than subsequent lines when used as an initial salvage treatment. On the other hand, a multicenter retrospective study found that CDCT was not inferior to HDCT as a salvage treatment, particularly in patients with IGCCCG low-risk.^[14]^

The primary tumor site, type of HDCT regimen, and best response to HDCT were independent prognostic factors for PFS. Einhorn et al^[13]^ identified IGCCG high-risk stage, platinum-refractory disease, and more or equal to third-line chemotherapy as independent prognostic factors. In 1996, Beyer et al^[16]^ conducted a multicenter study in 310 patients with relapsed/refractory GCT treated with HDCT and ASCT. The study found that PD before HDCT, primary mediastinal nonseminomatous GCT, refractory to initial cisplatin-based chemotherapy, and human chorionic gonadotropin levels of ≥ 1000 U/L before HDCT were independent prognostic factors. Interestingly, the primary tumor site and HDCT response rate appear to be common prognostic factors.

Even though there were no direct comparisons for different HDCT regimens in retrospective studies, similar response rates for CE, the most often used HDCT regimen, were obtained. Nonetheless, we found that carbo–PEC–taxol outperformed other regimens regarding PFS, including the CE regimen. In a retrospective study of 28 patients with relapsed/refractory GCT, Sharma et al^[18]^ evaluated the survival outcomes of patients who received carbo–PEC–taxol HDCT regimen as an initial salvage therapy. In this study, the median PFS was 17.3 months following a median follow-up period of 5 years. Although this study showed that HDCT has the potential for a long-term response, it lacked a control group to assess different HDCT regimens. In a study of 74 patients who had HDCT treatment with iphosphamide and CE, the 1-year CR was 19%, and 2 patients (3%) died due to treatment-related complications.^[19]^ Notably, 18% of patients in the same study had mediastinal involvement, and most of the patients received at least 2 lines of CDCT before HDCT. In a phase II study, the carboplatin–etoposide–thiotepa regimen was used after 3 cycles of TIP induction in 80 patients as salvage HDCT. The results showed that the 3-year OS was 30%, with only 1 patient (1.25%) dying from treatment-related pulmonary hemorrhage.^[20]^ Despite demonstrating a survival advantage for carbo–PEC–taxol, the size of our cohort, particularly in the TTC subgroup, limits us from making general conclusions. Multicenter studies with a predominance of patients with GCT who received HDCT are required to comprehensively evaluate the different types of HDCT regimens in this regard.

Because 90% of our patients did not receive post-HDCT treatment, we evaluated the risk factors affecting OS. The 2-year OS of our study was 45.7%; however, the 5-year OS data are not yet mature. The best response to HDCT was the only independent risk factor for OS. HDCT-related mortality for our patients was 10%. Treatment-related mortality ranged from 1.2% to 20% in previous studies evaluating HDCT efficacy.^[12,13,20]^ The HDCT-related mortality rate varies significantly among centers. Because of the increased mortality risk, clinicians should be cautious when performing HDCT on patients with brain metastases and bulky masses. Appropriate patient selection and treatment may improve survival outcomes in highly experienced centers.

The limitations of our study are the retrospective design and a small number of patients. We were unable to determine their prognostic value because laboratory parameters were unavailable. In addition, we were unable to determine the OS of specific subgroups due to the immature data. In conclusion, despite its mortality risk, HDCT with ASCT support effectively treats patients with relapsed/refractory GCT. Survival is indicated by the best response to HDCT and the primary tumor site. Furthermore, carbo–PEC–taxol may provide an additional survival benefit for patients with relapsed/refractory GCT.

## Author Contributions

FF: Methodology, Data curation, Writing—Original draft preparation, NP: Conceptualization, Validation, Data curation, NK: Methodology, Writing—Original draft preparation, AY: Validation, Writing—Original draft preparation, MAA: Conceptualization, Writing—Original draft preparation, ZG: Supervision, Validation, Methodology, MB: Supervision, Conceptualization, Methodology, Writing—Original draft preparation.

**Conceptualization:** Nail Paksoy, Melin Aydan Ahmed, Mert Basaran.

**Data curation**: Ferhat Ferhatoglu, Nail Paksoy.

**Methodology:** Ferhat Ferhatoglu, Nijat Khanmammadov, Zafer Gülbas, Mert Basaran.

**Supervision:** Zafer Gülbas, Mert Basaran.

**Validation:** Nail Paksoy, Anil Yildiz, Zafer Gülbas.

**Writing—original draft:** Ferhat Ferhatoglu.

**Writing—review & editing:** Ferhat Ferhatoglu, Nijat Khanmammadov, Anil Yildiz, Melin Aydan Ahmed, Mert Basaran.

## Supplementary Material


