# Supplementary material for: Therapeutic efficacy of high-dose chemotherapy with autologous stem-cell transplantation in 44 relapsed or refractory germ-cell tumor patients: A retrospective cohort study
Source: Medicine (Baltimore). 2024 Feb 23;103(8):e37213. doi: 10.1097/MD.0000000000037213 (PMC11309616; doi:10.1097/MD.0000000000037213)
Supplement: Supplementary file 1 [file medi-103-e37213-s001.docx]

| **The cause of death** | intracranial hemorrhage | intratumoral bleeding | intratumoral bleeding | intracranial hemorrhage |
| --- | --- | --- | --- | --- |
| **Time to death after HDCT (days)** | 11 | 7 | 26 | 16 |
| **HDCT regiment** | BEAM | CE | CE | Carbo-PEC-taxol |
| **HDCT line** | 3 | 4 | 3 | 4 |
| **Best response to first line treatment** | CR | IR | IR | IR |
| **Time to first relapse (months)** | 101,5 | 30.Ağu | 24,8 | 84,9 |
| **Primary tumor site** | Gonadal | Gonadal | Gonadal | Retroperitoneal |
| **Histology** | Seminoma | Non-seminoma | Non-seminoma | Non-seminoma |
| **Age** | 39 | 44 | 37 | 33 |
| **Patients** | Patient 1 | Patient 2 | Patient 3 | Patient 4 |

BEAM: BCNU-etoposide-cytarabine-melphalan; Carbo-PEC-taxol: Carboplatin-Etoposide-Cyclophosphamide-Paclitaxel; CE: Carboplatin-Etoposide; CR: Complete response; HDCT: High-dose chemotherapy; IR: Incomplete response.

**Supplementary Table 1: Clinical details of patients with HDCT-related death**
